# Alcohol preparation compared to traditional surgical hand antisepsis: acceptance by surgical team at a private hospital in Brazil

**DOI:** 10.1186/2047-2994-4-S1-P163

**Published:** 2015-06-16

**Authors:** JY Kawagoe, AR Toniolo, CV Silva, FG Menezes, M Hutter, P Zimmer, L Barbosa, L Correa, Camila Santos, Leonilda Pontes, Helena Castagna, Maria F Cardoso, Priscila Gonçalves

**Affiliations:** 1 Nursing Post-graduation Course, Albert Einstein Nursing Faculty, Sao Paulo, Brazil; 2 Infection Control Service, Hospital Israelita Albert Einstein, Sao Paulo, Brazil; 3 Infection Control Service, Albert Einstein Hospital, São Paulo, Brazil; 4 Surgical Center, Albert Einstein Hospital, São Paulo, Brazil; 5 GOJO, Sao Paulo, Brazil

## Introduction

Traditional surgical hand scrubbing (TSHS) has been replaced by alcohol-based formulation (ABF) in many countries due to antimicrobial efficacy, easy application, lower skin damage, time saver and no recontamination risk by rinsing hands with water [1]. In Brazil, chlorhexidine (CHG) and povidone-iodine (PVPI) are used for TSHS.

## Objectives

Compare one ABF to TSHS, evaluating surgical team acceptance and time, water, products and waste savings.

## Methods

This before-after intervention evaluation, quantitative study was conducted in two operating suites (26 rooms) at a 650-bed private hospital - São Paulo, Brazil. The ABF approved for surgical hand antisepsis by ASTM-E1115, CEN-EN 12791 and Brazilian Health Surveillance Agency was available for 12-week (May-July/2014). The subjects that met inclusion criteria answered two questionnaires: before and after ABF introduction, evaluating the following attributes: softness, dryness, irritation, skin feel, easiness in putting on surgical gloves, drying time, etc. Chi-square and McNemar tests were used. Time and water consumption were also measured.

## Results

52 (55.9 %) subjects completed the study. The majority were male (34; 65.4%), age of 36–55 years and > 16 years performing surgery (31; 59.6%). ABF was considered excellent/good by 47 (90.4%). Procedure simplifier/time saver, good fragrance, soft/comfortable/hydrated hands skin, non-abrasive/non-irritating were evaluated positively, p < 0.001. There were per person/hand antisepsis: approximately 73 seconds’ time saving, an average of 33.1 liters of water consumption saving and waste saving (surgical brushes/sterile towels).

## Conclusion

The ABF for surgical hand antisepsis had an excellent/good acceptance by the members of surgical team. It results in considerable savings in water and healthcare associated residues as the environment is concerned.

## Disclosure of interest

J. Kawagoe Grant/Research support from: GOJO - Provision of alcoholic products and techinical and administrative support, A. Toniolo: None declared, C. Silva: None declared, F. Menezes: None declared, M. Hutter: None declared, P. Zimmer: None declared, L. Barbosa Employee of: GOJO - Provision of alcoholic products and techinical and administrative support, L. Correa: None declared.
